# Regional classification of high PM_10_ concentrations in the Seoul metropolitan and Chungcheongnam-do areas, Republic of Korea

**DOI:** 10.1007/s10661-023-11732-6

**Published:** 2023-08-24

**Authors:** Woosuk Choi, Min Young Song, Jong Bum Kim, Kwanchul Kim, Chaeyoon Cho

**Affiliations:** 1 Department of Data Science, Sejong University, Seoul, Republic of Korea; 2 Division of Climate and Environmental Research, Seoul Institute of Technology, Seoul, Republic of Korea; 3 Seohaean Research Institute, ChungNam Institute, Hongseong, Republic of Korea; 4 Advanced Institute of Convergence Technology, Seoul National University, Gyeonggi-Do, South Korea; 5 Research Center for Transportation, Climate, and Environment, Hyundai Insurance, Seoul, Republic of Korea

**Keywords:** PM_10_, Metropolitan area, Clustering analysis, Regional classification, Air quality management

## Abstract

Since the Seoul metropolitan area is a highly developed megacity, many people are often exposed to high concentrations of particulate matter (PM), with mean aerodynamic diameters equal to or less than 10 μm (PM_10_), in cold seasons. PM_10_ concentrations can be influenced by a combination of various factors, including meteorological conditions, anthropogenic emissions, atmospheric chemical reactions, transboundary transport, and geographic characteristics. However, the establishment of an efficient air quality management plan remains challenging due to the limited understanding of the regional PM concentration characteristics. Here, the Seoul metropolitan (Seoul, Gyeonggi-do, and Incheon) and Chungcheongnam-do (Chungcheongnam-do, Daejeon, and Sejong) areas were regionally classified to identify the spatiotemporal air quality in areas where megacities and emission sources are mixed. The four representative regions were determined using the K-means clustering method based on the temporal variations in the observed PM_10_ concentrations. The first cluster consisted of small cities in the southern and eastern parts of Gyeonggi-do and Chungcheongnam-do, respectively, while the second cluster consisted of Incheon, West Gyeonggi-do, and Seoul. In addition, the third and fourth clusters included West Chungcheongnam-do and East Gyeonggi-do, which are adjacent to the Yellow Sea and downstream area of the westerly wind, respectively. The characteristics of each cluster during the high PM_10_ concentration events are explainable by wind patterns and the local air pollutant emissions, including nitrogen oxides and sulfur oxides. The obtained regional classification was different from the provincial-level administrative division of South Korea. Therefore, the present study is expected to be a scientific basis for overcoming the limitations of air quality management in administrative districts.

## Introduction

The suspended particulate matter (PM), with mean aerodynamic diameters equal to or less than 10 μm (PM_10_), causes various environmental and health problems, such as limited visibility and respiratory diseases (Dockery et al., 1993; Kim et al., 2020, 2022a; Pope & Dockery, 2006). It has been known that high concentrations of 24-h mean PM_10_, exceeding 100 μg m^−3^, occur over 20 days during the cold season in the Korean Peninsula (Ku et al., 2021; Lee et al., 2011). Indeed, several factors causing high PM concentrations in the Korean Peninsula have been reported. Among them, transboundary transport from the western region of Korean Peninsula by mid-latitude westerly winds and nearby industrial emissions have been widely studied for identifying high PM episodes (Bae et al., 2019; Choi et al., 2019; Jeong et al., 2011; Kim et al., 2018; Lee et al., 2011, 2013, 2019a, b, 2021; Park et al., 2021; Pouyaei et al., 2022). Another major factor causing high PM concentrations is the synoptic pattern of atmospheric stagnation that blocks the outward propagation of aerosols (Cho et al., 2021, 2022; Jeon et al., 2019; Jeong & Park, 2017; Jung et al., 2019; Kim et al., 2017a, b, c; Shin et al., 2021; Yun & Yoo, 2019). Moreover, the impacts of secondary aerosol formation have been known to contribute considerably to the air quality deterioration (Jordan et al., 2020; Sun et al., 2013, 2016; Xu et al., 2022). Given the harmful effects of PM on human health, potentially significant damages are expected in densely populated areas.

The Seoul metropolitan area, including Seoul, Gyeonggi-do, and Incheon, is a megacity in South Korea (Ho et al., 2021). Therefore, various types of air pollutants can be emitted from urban sources, such as residences, vehicles, coal combustion, and usage of organic solvents. Indeed, there are many large-scale coal-fired power plants in Chungcheongnam-do in the southern neighboring region of the Seoul metropolitan area, including Dangjin, Seosan, Boryeong, Taean, and Seocheon, as well as nearby steel mills and petrochemical complexes. Emitted air pollutants from these coal-fired power plants could alter the chemical composition and variability of PM through gas-to-particle conversion in the atmosphere (Cho et al., 2019; Choi et al., 2021; Kim et al., 2017a, b, 2022b; Lee et al., 2015; Vellingiri et al., 2015). Most regional industry-related air quality management policies are implemented by the administrative districts. However, districts are defined by artificial boundaries (or borders) that do not typically reflect topography, airflow, and emission sources of PM. Therefore, coordination among several districts to develop regional air quality management and strategy have not been carried out in consideration of regional variations in PM concentrations.

In this study, the regional air quality in the Seoul metropolitan and Chungcheongnam-do area was classified based on the temporal variations in the PM_10_ concentrations. There are many industrial and residential emissions, and dense population (~ 30 million) in study area, so great health and social damages due to exposure are expected. Hence, diagnosis of regional PM_10_ variability in target area is required. Numerous factors could be comprehensively considered in the clustering analysis of PM_10_ concentrations, including local emissions, airflow transport, topographic effects, and atmospheric chemical reactions. In fact, this regional classification could be essential information not only to understand the mechanism of high PM_10_ concentrations but also to examine the factors controlling the regional air quality (Stolz et al., 2020). To investigate the causes of the differences in the PM_10_ concentrations by region and to assess the reliability of regional classification, representative cases of high PM_10_ concentrations were studied.

## Materials and methods

### Data

In this study, the observed PM_10_ concentration data from a total of 87 sites, representing each municipality in the Seoul metropolitan area (Seoul, Gyeonggi-do, and Incheon) and Chungcheongnam-do region (Chungcheongnam-do, Daejeon, and Sejong), were obtained from AirKorea website (https://www.airkorea.or.kr/). Measures at each site and monitoring network system are managed by the Ministry of Environment, Korea Republic. The PM_10_ concentrations were measured using the *β*-ray attenuation instrument (e.g., Met One BAM-1020 or Thermo Anderson FH 62 C14 Series; Lee et al., 2006). The basic principle of *β*-ray attenuation is that suspended particles with a diameter of 10 µm or less in the air were first collected on a filter for at 1-h intervals, then the intensity of the absorbed or dissipated *β*-ray emitted from the atmospheric *β*-ray light source was measured by a sensor as it passes through the aerosol-collected filter (Chang & Tsai, 2003). The PM_10_ concentrations can be continuously deduced using the difference between the absorbed and dissipated *β*-ray intensities. Because the high PM_10_ concentrations frequently occur in cold seasons (Lee et al., 2011), the observed PM_10_ concentration data in 5 months from November to March over the 2018–2021 period, representing a total of three cold seasons, were used in this study for clustering analysis. Although this is an hourly dataset, their normalized daily averages, with an average and standard deviation of 0 and 1, respectively, were used to evaluate the temporal variability rather than the difference in the absolute PM_10_ concentrations according to the observatories.

In addition, hourly wind directions and speed, air temperature, and air humidity data were obtained from the Korea Meteorological Administration to assess the regional variations in the PM_10_ concentrations. In total, three surface weather stations, namely Boryeong (36.33° N, 126.56° E), Seoul (37.57° N, 126.97° E), and Yangpyeong (37.49° N, 127.49° E), were considered in this study. Observed data during the periods of high PM_10_ concentrations (see “Characteristics of the four clusters during the high PM_10_ concentration episodes” below) were investigated to reveal the regional PM_10_ variability in the study area.

### Methodology

In this study, a clustering technique is applied to group together stations that show similar temporal PM_10_ concentration variations. The *K*-means clustering method is one of the representative unsupervised machine learning algorithms (Steinley, 2006). First, a *K* number of centroids is randomly defined in the dataset to be classified. In this method, each data is classified into the group of the closest centroids based on the Euclidean distance. Second, new centroids are set by calculating the average of the data assigned to each cluster. This process repeatedly determines the centroid of each cluster by calculating the distance between the datasets and classifies the representative groups until achieving slight changes in the cluster centroids.

The distance between datasets in the *K*-means clustering analysis is usually calculated based on the Euclidean distance, representing the difference between vector elements of each object at the same time. However, the Euclidean distance calculation may not be able to appropriately reflect data characteristics if the dataset includes time dimension (e.g., time series data) even under similar characteristics of objects at different time steps. Therefore, the dynamic time warping (DTW) method, connecting the points with the shortest distances by considering the speed of each time series data, was used in this study (Aach & Church, 2001; Aghabozorgi et al., 2015; Petitjean et al., 2011; Suris et al., 2022). Indeed, this method calculates the distance between two given temporal datasets, with sequential alignment (time = *t*), and the minimum distances of three neighboring time step combinations, namely (*t-1*, *t*), (*t*, *t-1*), and (*t-1*, *t-1*), of the first and second array. By minimizing the sum of the distances, the DTW can be performed easily to analyze temporal sequences, such as audio and graphic data, resulting in an optimal match by reflecting different time-evolving data. Therefore, the DTW method can be used to classify different datasets within the same cluster in the case of time lags between them according to their variability.

The number of clusters (*K*) can exhibit a deterministic impact on the cluster analysis results. Although *K* can be determined subjectively by researchers and according to the purposes of studies, the elbow method was used in this study to find the optimal *K* number (Kodinariya & Makwana, 2013). This method uses the *K*-value of the inflection point for determining the distortion scores (i.e., cost function), which is the sum of distances from the centroid of each cluster that rapidly decreases with increasing the number of clusters.

To understand comprehensively the dynamic mechanisms controlling the three representative high-PM_10_ concentration cases, backward trajectories were analyzed using the Hybrid Single-Particle Lagrangian Integrated Trajectory (HYSPLIT) model (Stein et al., 2015). The model results were calculated by considering the Global Data Assimilation System (GDAS) as meteorological input data. These data were, in fact, obtained from the National Oceanic and Atmospheric Administration website (http://www.arl.noaa.gov/HYSPLIT_info.php). The 72-h back trajectories initialized 1000 m above ground level (a.g.l.) at each site were analyzed to trace the air mass movement in this study.

## Results

### Regional classification of PM_10_ according to its concentration variability

**Fig. 1:**
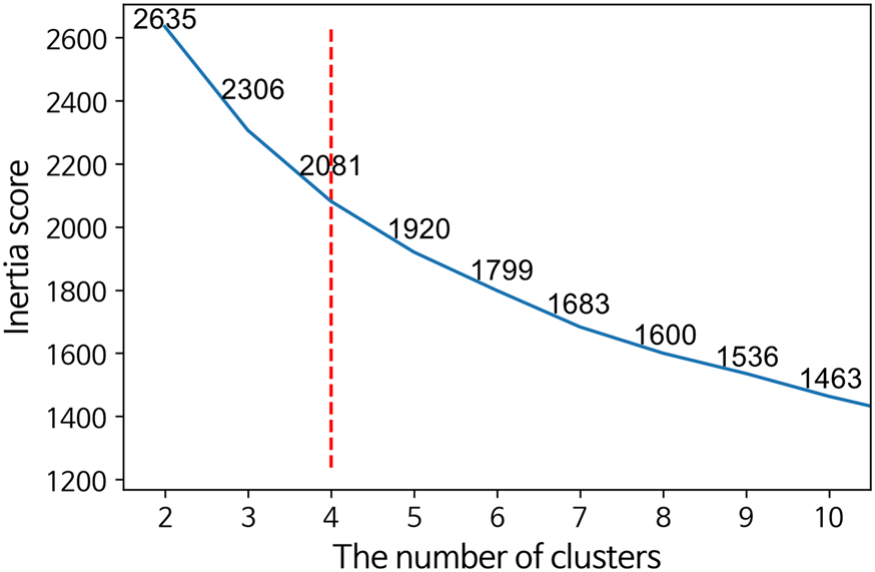
Inertia scores of K-means clustering according to the number of clusters

Considering the distortion scores as a function of the number of clusters (Syakur et al., 2018), it was found that the elbow point was four (Fig. 1). Therefore, PM_10_ variabilities in the Seoul metropolitan and Chungcheongnam-do area were classified into four representative groups. In general, clustering analysis is highly dependent on the dataset, as it finds representative patterns from the original data. However, the regional distributions of the four clusters were similar, even if the analysis was performed separately according to each winter season (not shown). Hence, this regional classification is considered a strong and clear result.

**Fig. 2:**
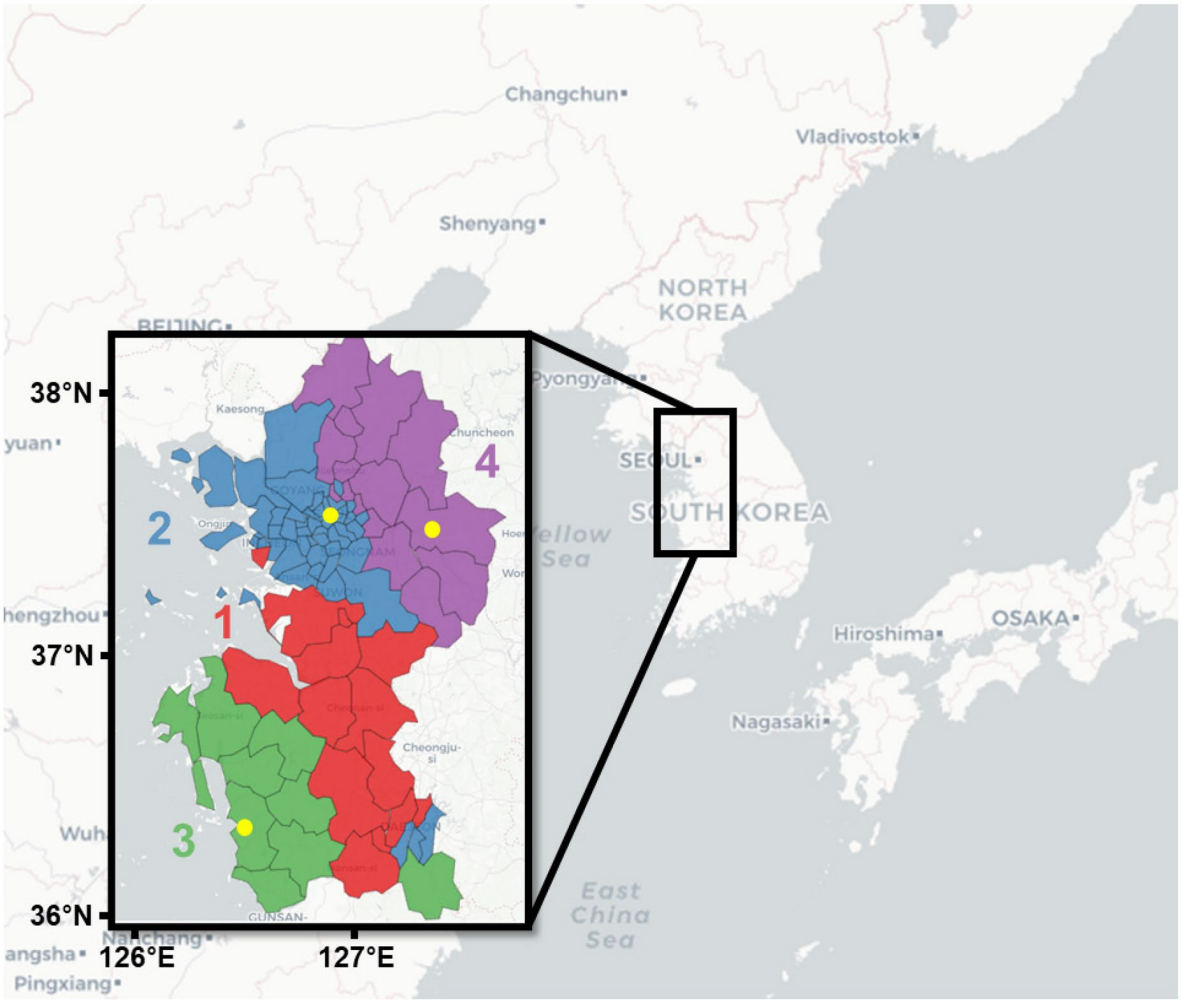
Classification of the representative regional clusters shown in different colors according to the PM_10_ concentration variations in the capital and surrounding regions of the Republic of Korea. Regions within clusters 1, 2, 3, and 4 are shown in red, blue, green, and purple areas, respectively. Yellow dots in clusters 2 (blue), 3 (green), and 4 (purple) represent Seoul, Boryeong, and Yangpyeong weather stations, respectively

Cluster 1 was observed mainly in the southern and northeastern parts of Gyeonggi-do and Chungcheongnam-do, respectively (red area in Fig. 2). In this area, aerosols are emitted from small industrial facilities, as well as from active movements of ships and vehicles in ports and highways, respectively. Regions of cluster 2 are located in the western parts of Gyeonggi-do, Seoul, and Incheon (blue area in Fig. 2). Since the Seoul metropolitan area is a largely populated megacity, regions within cluster 2 are expected to be affected by emissions of PM from urban activities, such as residential and traffic-related activities. Cluster 3 was mainly observed in the western part of Chungcheongnam-do, bordering the Yellow Sea. Since several large-scale coal-fired power plants are present in this area (e.g., Dangjin, Seosan, Boryeong, Taean, and Seocheon coal-fired power plants), local emissions may influence greatly air quality in this region. It should be noted that small blue and green areas in the southeastern part suggest a kind of noise for clustering analysis. Cluster 4 was observed in the eastern part of Gyeonggi-do. Although there were few local emission sources in cluster 4, pollutants emitted from the western part are expected to impact the air quality in this area. Since westerly and northwesterly winds are climatologically predominant in the Korean Peninsula during cold seasons, all regions could be also influenced by transboundary aerosol transport from other nations (Bae et al., 2019; Kim et al., 2018; Lee et al., 2013; Pouyaei et al., 2022). Overall, representative clusters of the PM_10_ variability were classified in these four regions based on various influencing factors, such as local emissions, transport, and secondary generation of PM (Nault et al., 2018; Park et al., 2022).

**Fig. 3:**
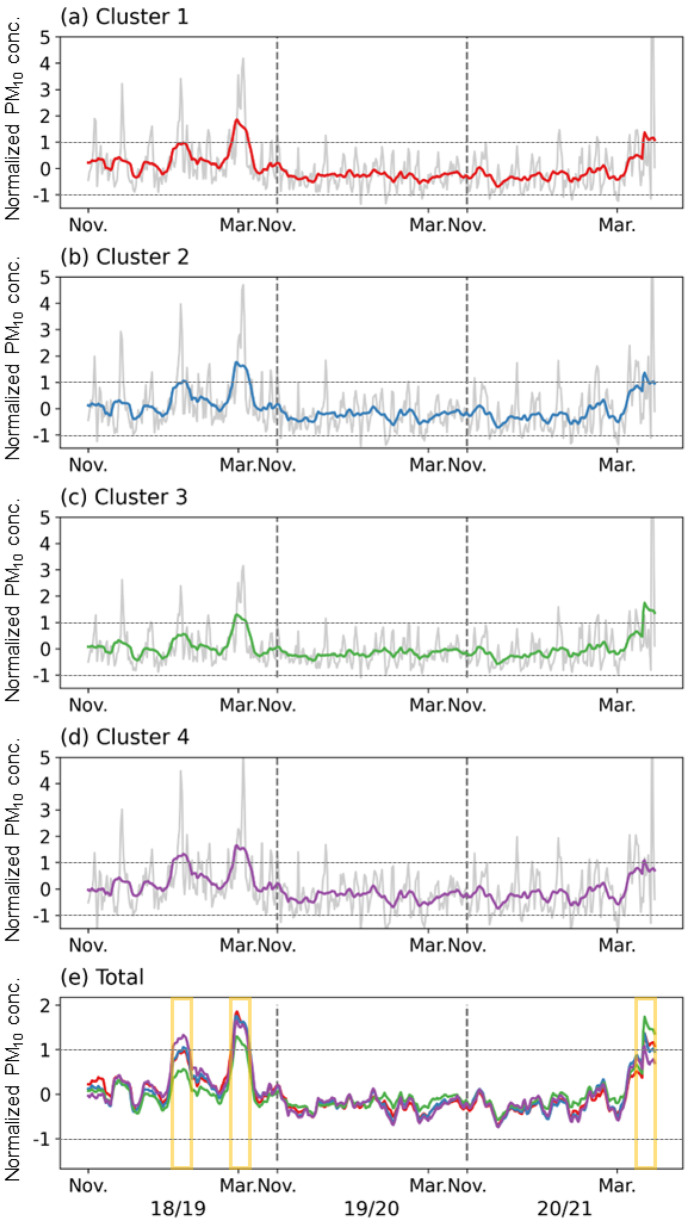
**a**, **b**, **c**, **d** Time series of the normalized PM_10_ concentrations in the four defined clusters in the November–March period of the three cold seasons. Gray and colored lines indicate representative concentrations within each cluster and their 15-day moving averages, respectively. **e** Four moving average lines are shown in **a**, **b**, **c**, **d**. The yellow-boxed periods in **e** correspond to 2 days of the three high PM_10_ concentration episodes

The variation in the normalized PM_10_ concentration within each cluster was examined in this study (Fig. 3). The data from November to March in boreal winter were connected and analyzed to represent three winters in a single time series. Since time series data were normalized for the entire analysis period based on the observation sites, most values were within the range of 0 ± 1. The variations in the PM_10_ concentrations in the four clusters were generally similar (Fig. 3e) because the study area (the Seoul metropolitan and Chungcheong area) is smaller than the synoptic scale (~ 1000 km) (Fig. 2). Therefore, it can be expected that the weather and external influences in this area would not be significantly different. However, the PM_10_ variability showed distinct patterns in specific periods of the time series. Mid-January 2019, early-March 2019, and late-March 2021 were selected as representative high PM_10_ concentration periods, during which the normalized PM_10_ concentrations increased, with one or more clusters exceeding a value of 1. However, they exhibited different levels between clusters (yellow shades in Fig. 3e). The characteristics of the regional concentrations during these three periods were further examined in this study.

**Fig. 4:**
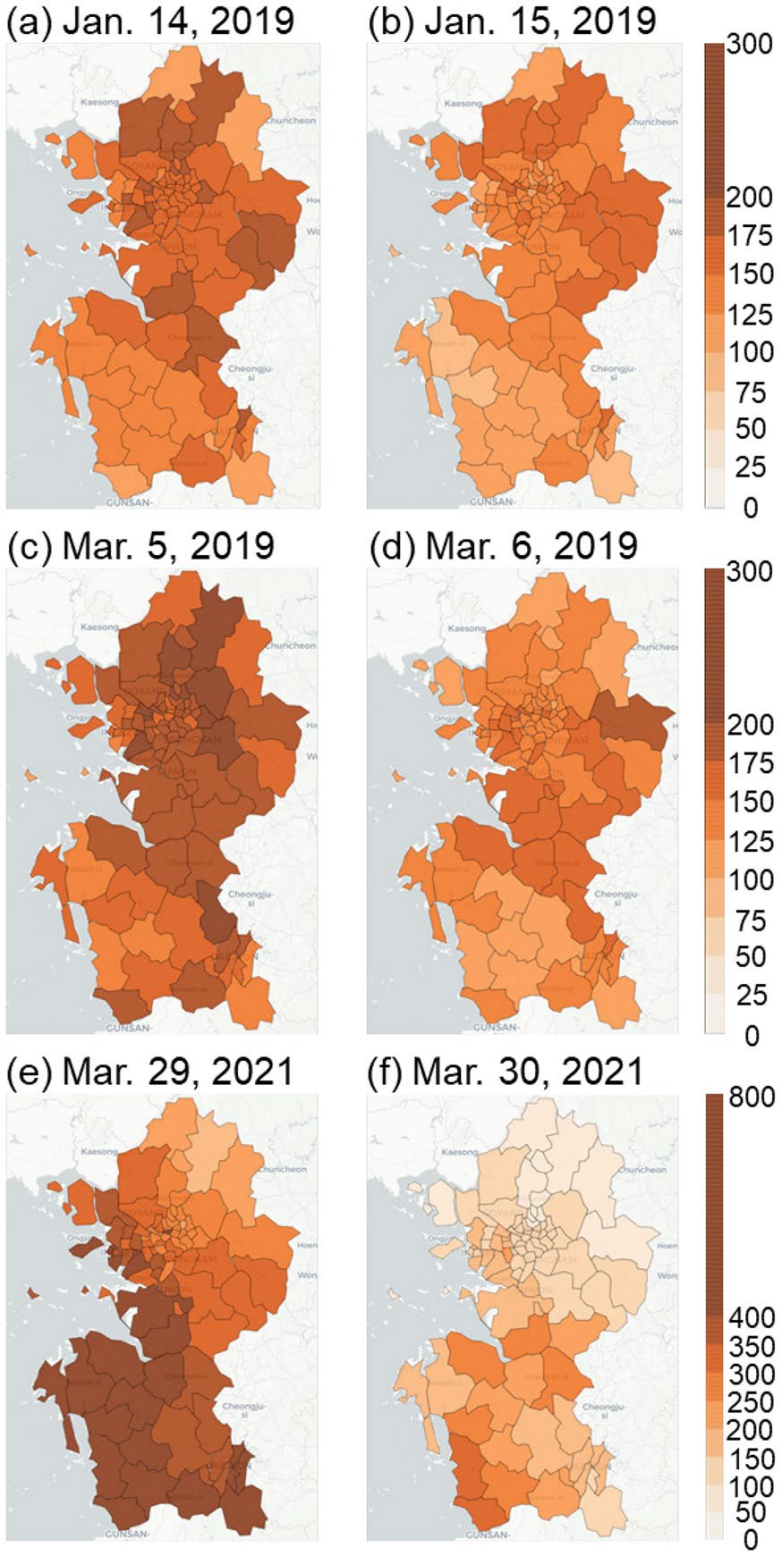
Spatial distributions of the average daily PM_10_ concentrations (μg m^−3^) in the three high PM_10_ concentration episodes

Among the three representative high PM_10_ cases, the regional average PM_10_ concentrations were observed on January 14–15, 2019, March 5–6, 2019, and March 29–30, 2021, when the differences in concentrations between clusters were the greatest (Fig. 4). The first case, in January 2019, was a well-known high PM concentration episode (Oh et al., 2020). The regional PM_10_ concentrations of cluster 3 in January 2019 were relatively low, while those of cluster 4 were relatively high (Fig. 3e). This finding was in line with the observed spatial distribution of PM_10_ concentrations, showing a PM_10_ concentration range of about 100–150 μg m^−3^ on January 14, 2019, in the western part of the Chungcheongnam-do area, next to the Yellow Sea. However, most eastern areas of Gyeonggi-do exhibited PM_10_ concentrations over 150 μg m^−3^ (Fig. 4a). Although the overall air quality was cleaner on January 15, 2019, than the day before, the same aforementioned regional differences were observed (Fig. 4b).

High concentrations of about 200 μg m^−3^ were observed on March 5, 2019, over most of the study area due to a well-known long-lasting haze episode (Shim et al., 2022). However, areas of cluster 3 showed a lower concentration range of 100–150 μg m^−3^ compared to those in other areas (Fig. 4c). This spatial distribution was, indeed, similar to that observed on the next day (March 6, 2019). Although the overall high concentration event was slightly attenuated, relatively low concentrations were also found in the western part of Chungcheongnam-do (Fig. 4d). The average daily PM_10_ concentration was up to 800 μg m^−3^ in the western coastal area of Chungcheongnam-do on March 29–30, 2021, which is inconsistent with the previous cases (Fig. 4e and f). In contrast, the observed PM_10_ concentrations were lower than 400 μg m^−3^ in most parts of Gyeonggi-do and Seoul, which is approximately half of that observed in Chungcheongnam-do (Fig. 4e). This regional distribution pattern was also observed on March 30, 2021, showing local high PM_10_ concentrations in Chungcheongnam-do (Fig. 4f). Although the study region is a small area, there were some regional differences in the PM_10_ concentrations differences during the high concentration period.

### Characteristics of the four clusters during the high PM_10_ concentration episodes

**Fig. 5:**
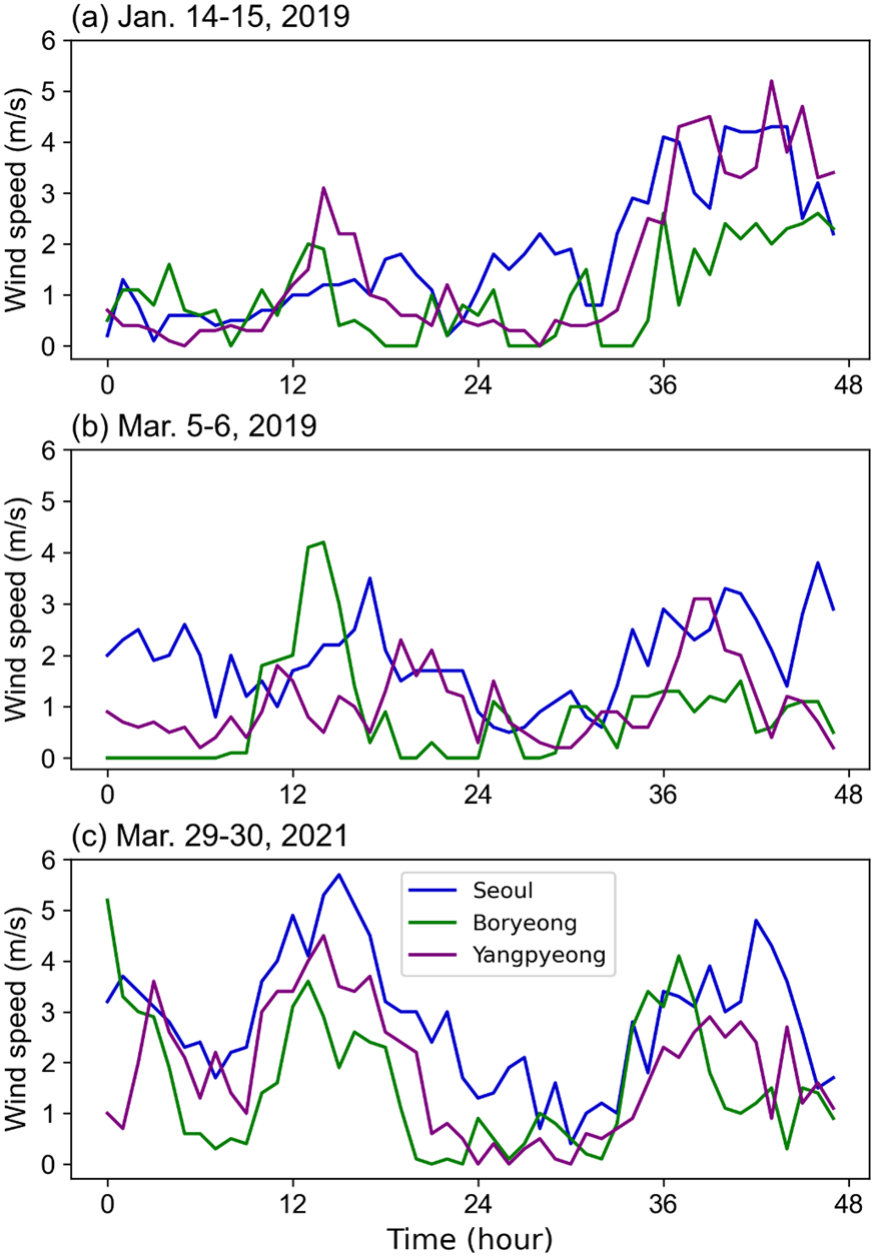
Time series of wind speed at the Seoul (blue), Boryeong (green), and Yangpyeong (purple) weather stations during the **a** January 14–15, 2019, **b** March 5–6, 2019, and **c** March 29–30, 2021

The characteristics of clusters 2, 3, and 4, showing distinct PM_10_ concentrations in the three cases, were further investigated in this study. To examine the characteristics of each cluster, the time series of surface wind speeds were examined according to each high PM_10_ concentration episode in Seoul (the representative point of cluster 2), Boryeong (cluster 3), and Yangpyeong (cluster 4) (yellow dots in Fig. 2). These sites are manned observatories, reflecting well the PM_10_ concentration variability in each cluster. Observatory sites in these three high PM_10_ concentration cases were located in areas where environmental conditions were likely to increase PM_10_ concentrations due to aerosol accumulation through atmospheric stagnation, with slight hourly wind speeds. Indeed, winds of 1–2 m s^−1^ were dominant on January 14–15 and March 5–6, 2019 (Fig. 5a and b). On March 29–30, 2021, winds of 2–4 m s^−1^ prevailed in three sites (Fig. 5c). Overall, a condition of general atmospheric stagnation in winter is constructed. Moreover, the obtained clustering results represented well the general characteristics of the winter air quality.

**Fig. 6:**
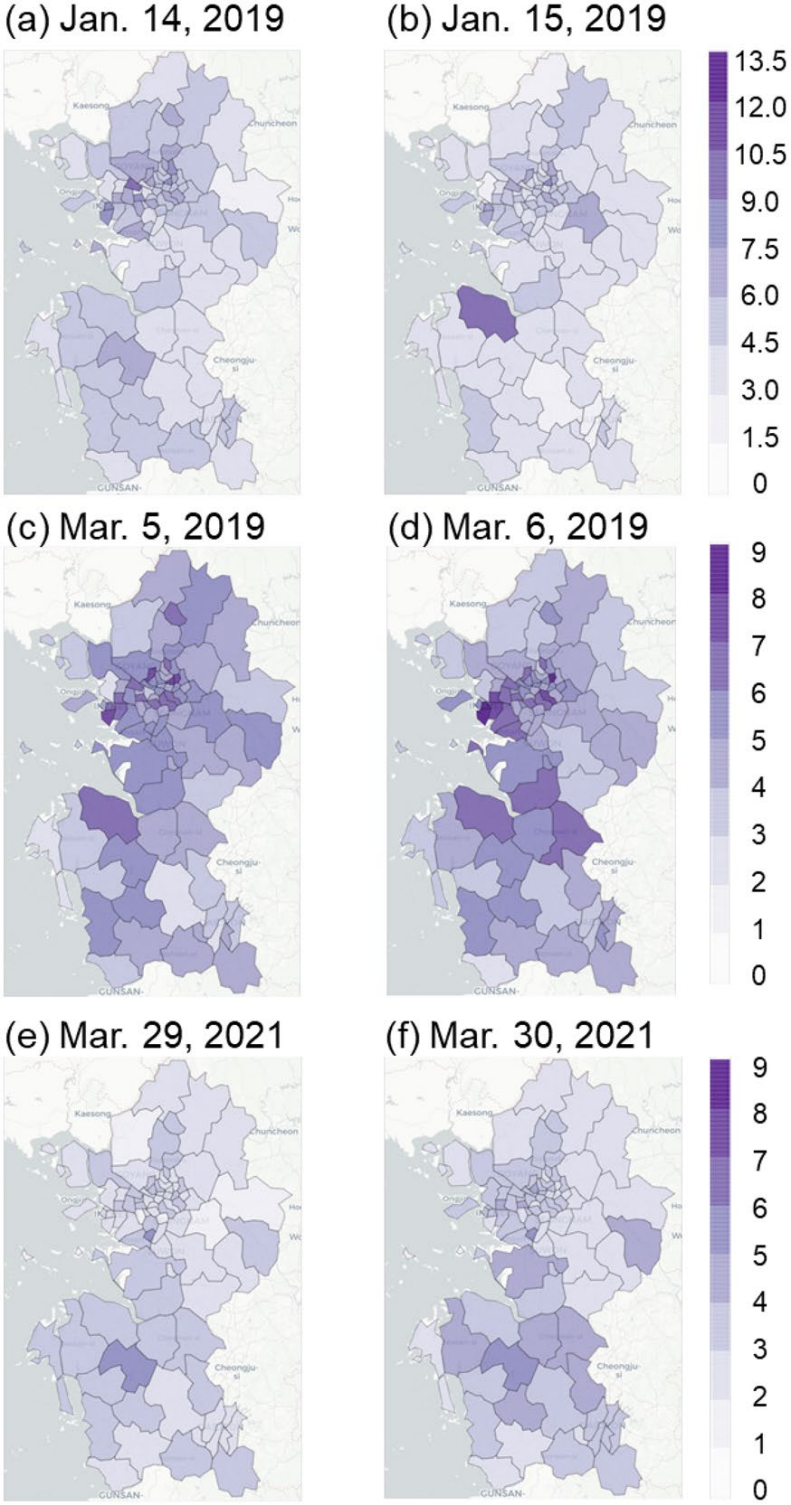
Spatial distributions of mean daily SO_2_ concentrations (10^−3^ ppmv) in the three high PM_10_ concentration episodes

As a gaseous precursor of aerosol, distributions of SO_2_ concentrations by region are investigated for each high PM_10_ concentration case (Fig. 6). Considering that the SO_2_ concentration on a typical day with low PM_2.5_ concentration is about 2**–**4 (10^−3^ ppmv), the SO_2_ concentration was also generally high in three representative cases. The SO_2_ concentrations in January 2019 were relatively high in the western part of Chungcheongnam-do (cluster 3) and the Seoul metropolitan area compared to other region (Fig. 6a and b). In addition, extremely high SO_2_ concentrations were observed in Dangjin (Fig. 6b), which might be due to the presence of a large-scale coal-fired power plant located in this area. High SO_2_ concentrations were also observed in March 2019 in the western part of Chungcheongnam-do, including Dangjin, Incheon, and some areas of Seoul (Fig. 6c and d). Sulfur oxides (SOx) may also be emitted from small factories located in the southern part of Gyeonggi-do (cluster 1). On the other hand, the measured SO_2_ concentrations in March 2021 were relatively high in the western part of Chungcheongnam-do, including Yesan County. In contrast, low SO_2_ concentrations were observed in the Seoul metropolitan area (Fig. 6e and f). Although there was no major emission source in Yesan, it was reported that PM from the coal-fired power plant located near the coastal area often emitted into Yesan, where they are blocked by the mountain ranges, thereby leading to high PM_2.5_ concentrations (Gong et al., 2021; Nam et al., 2018).

**Fig. 7:**
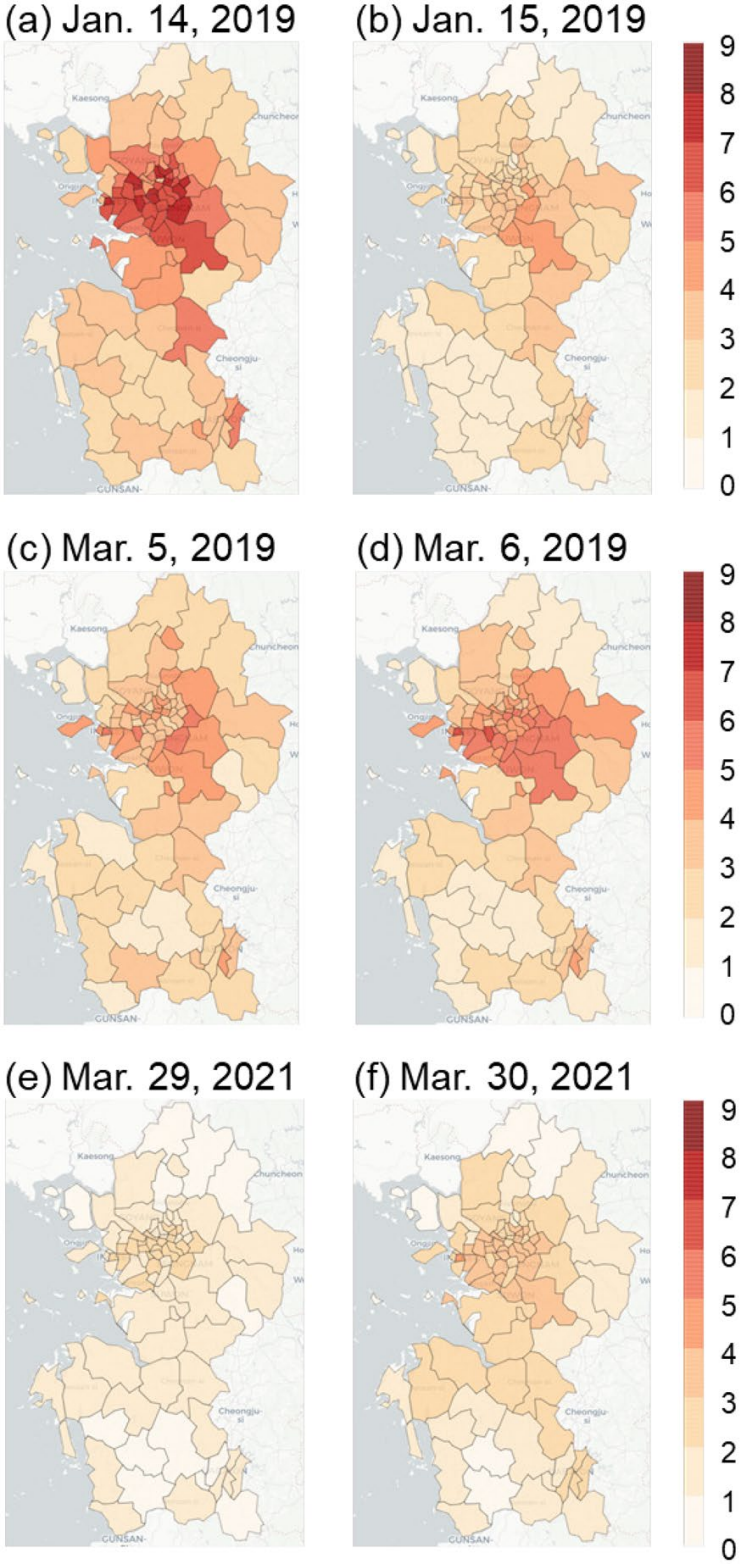
Spatial distributions of the mean daily NO_2_ concentrations (10^−2^ ppmv) in the three high PM_10_ concentration episodes

Similar to SO_2_, the distributions of the NO_2_ concentrations in the three representative cases were investigated (Fig. 7). Given the NO_2_ concentrations are 1**–**2 (10^−2^ ppmv) during the normal clean day, relatively high NO_2_ concentrations were observed in the case of January 2019 in Seoul and neighboring areas, while low NO_2_ concentrations were observed in Chungcheongnam-do (Fig. 7a and b). Since there were no major emission sources in the region of cluster 4 and there were significant aerosol precursor emissions in Seoul metropolitan area (Fig. 7a and b), atmospheric chemical reactions might be the main mechanism driving the air quality deterioration in regions of cluster 4. These patterns of high and low concentrations of NO_2_ in the Seoul metropolitan area and Chungcheongnam-do, respectively, were similar in March 2019 (Fig. 7c and d). In contrast, although the observed NO_2_ concentrations in the metropolitan area in March 2021 were relatively higher than those observed in the other regions, they were generally lower than the other high PM_10_ concentration cases (Fig. 7e and f).

**Fig. 8:**
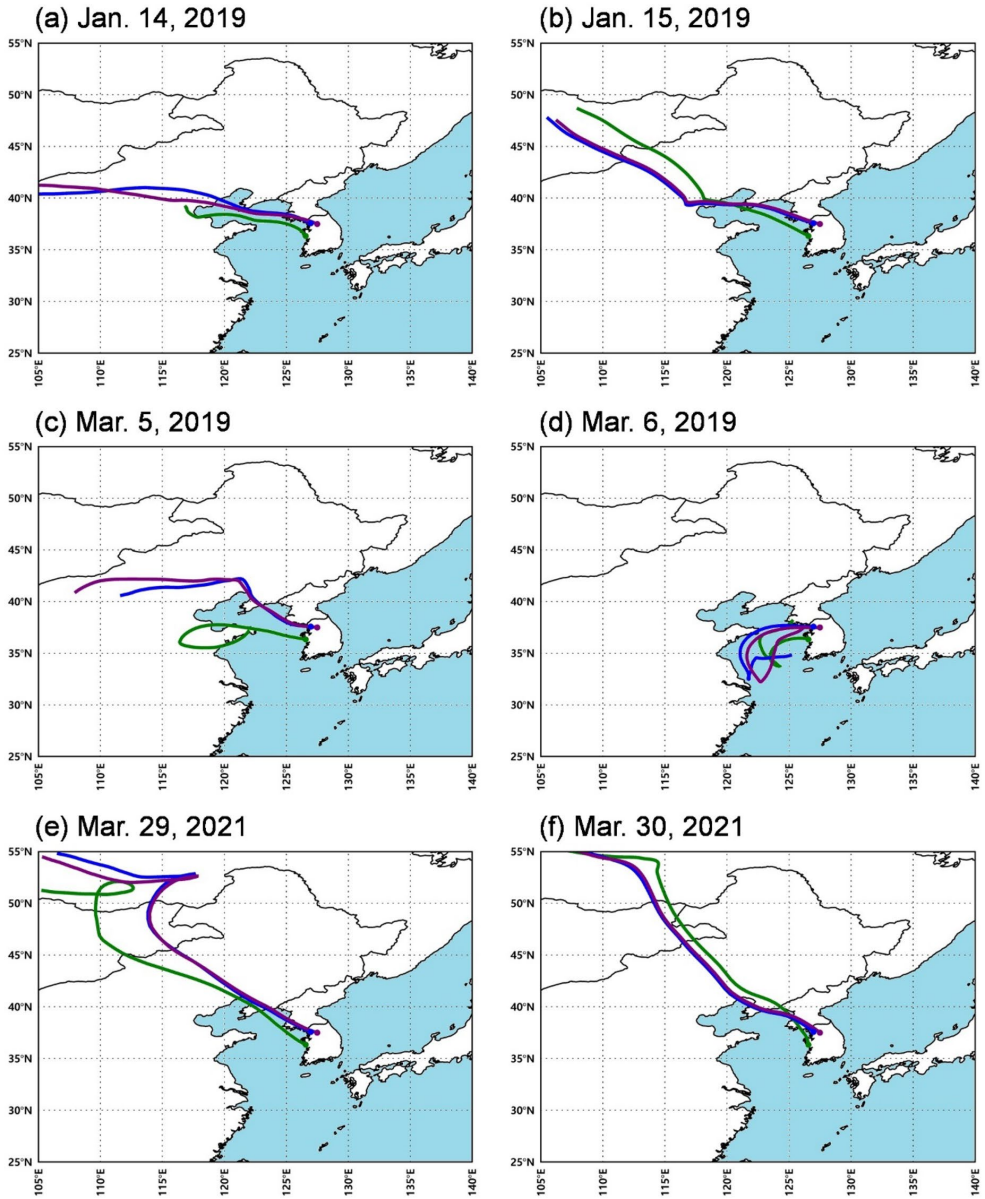
72-h back trajectories at the Seoul (blue), Boryeong (green), and Yangpyeong (purple) weather stations initialized at 00:00 UTC in the three high PM_10_ concentration episodes at 1000 m above ground level

The HYSPLIT model was used to analyze the air inflows at three representative stations in Seoul, Boryeong, and Yangpyeong. In the case of January 2019, the air masses at the three sites were transported over a long distance from China’s mainland (Fig. 8a and b). In March 2019 case, the long-distance air inflow from Beijing-Tianjin-Hebei to Seoul and Yangpyeong increased the PM_10_ concentrations. However, Boryeong was affected by the nearby air mass from the Yellow Sea during the previous two days (Fig. 8c and d). The results of the back trajectories showed a typical yellow dust pathway in March 2021 as airflow from the Gobi Desert in Mongolia (Fig. 8e and f). Overall, it is clear that the regional PM_10_ concentrations were modulated by the local influences of atmospheric stagnation and long-distance transportation from China’s mainland.

## Discussion

To further discuss the variation in the PM_10_ concentrations within each cluster, chemical factors that can lead to secondary particle formation were analyzed with respect to the wind directions and speeds. To ensure a comprehensive analysis, major aerosol precursors, such as nitric acid (HNO_3_) and sulfuric acid (H_2_SO_4_), should be considered in the investigation. Since ion components are not often measured in observatories, the regional SO_2_ and NO_2_ concentrations in sulfur and nitrogen oxides, respectively, were considered in this study as alternatives since they are measured at all times like PM_10_. SO_2_ and NO_2_ can be converted into strong acids, namely, H_2_SO_4_ and HNO_3_, by reacting with H_2_O and O_3_ in the atmosphere, respectively. Subsequently, these aerosol precursors can be combined with ammonia (NH_3_) in the atmosphere to form particles, such as ammonium sulfate ((NH_4_)_2_SO_4_) and ammonium nitrate (NH_4_NO_3_) (Bauer et al., 2007; Wang et al., 2005). Sulfur oxides in the study area are mainly emitted from coal-fired power plants, especially from the Dangjin plant, while traffic-related emissions are the major source of nitrogen oxides. In addition, NH_3_ might be released from livestock farms in Chungcheongnam-do and agricultural lands in the Gyeonggi-do plains. It is, therefore, necessary to discuss the aerosol concentration variabilities, taking into account the locations and types of the emission sources.

In the three abovementioned cases, the SO_2_ and NO_2_ concentrations, as well as wind transport, were comprehensively analyzed to reveal the characteristics of the PM_10_ concentration variability within each cluster. Since the SO_2_ concentrations in the Seoul metropolitan area were similar to those observed in Chungcheongnam-do in January and March 2019, it is assumed that the contribution of NO_2_ to the high PM_10_ concentration in the Seoul metropolitan area might be high if the regional differences between the direct aerosol emissions and transboundary transport are not substantial. Although there were not many major emission sources in the region of cluster 4, high PM_10_ concentrations are often derived from air pollutant inflows from Incheon, Seoul, or North Korea, considering the prevailing northerly and westerly winds. The prediction accuracies of the air quality by subregions may be improved by considering the regional PM_10_ concentration characteristics of this study in existing air quality prediction models.

It should be noted that the Korea Meteorological Administration (https://www.weather.go.kr/weather/asiandust/observday.jsp?type=2&stnId=108&year=2021&x=5&y=6) reported yellow dust over the Korean Peninsula on March 29–30, 2021 (Fig. 8e and f). The SO_2_ concentrations in this period were relatively high in the western part of Chungcheongnam-do. However, it was difficult to interpret the variations in the PM_10_ concentrations based on SO_2_ and NO_2_ due to the large-scale transportation of the yellow dust. Although yellow dust from China flowed into the Korean Peninsula and increased the PM_10_ concentrations, its regional distribution might differ due to topography, location, and various environmental conditions of each district.

To further investigate the secondary formation of aerosols and their effects on human health, it would be better to analyze the PM concentration data, with mean aerodynamic diameters of ≤ 2.5 μm (PM_2.5_), which are smaller particles than PM_10_, making it possible to comprehensively interpret the aerosol effects on human health, including respiratory diseases. However, the PM_2.5_ concentration dataset is not suitable for multiple station analysis because there are much more missing values than PM_10_ concentrations. At each monitoring station, concentrations of PM_2.5_ and PM_10_ are independently observed at the same time. If observed PM_2.5_ concentration is higher than PM_10_ (e.g., mainly on clean days), observation of PM_2.5_ is considered incorrect and treated as missing, resulting in more missing values of PM_2.5_ dataset. Hence, the PM_2.5_ concentrations dataset was not appropriate input data for training machine learning algorithms, limiting further discussion of the coarse fractions (i.e., PM_10_–PM_2.5_) and contributions of PM_2.5_ to the PM_10_ masses. A comprehensive analysis may be possible if the operational measure of PM_2.5_ is stabilized, with more recorded data, in the future. Moreover, considering PM and gas phases of various air pollutants in the classification of the regional characteristics of air quality in future studies may be of great significance for improving air quality and maintaining population health through national policies. More clear statements will be possible in the future by numerical predictions of PM_2.5_ concentrations without missing data and various air pollutants relevant to secondary particle formation.

The present study provides further insights into air pollution issues in other megacities worldwide suffering from air pollution problems. Indeed, low-emission zones have been implemented in many cities to reduce traffic volumes in urban areas. In addition, the use of renewable energies, which do not emit PM, such as solar and wind power generation, has been promoted. The air pollution characteristics may vary according to the geographical, climatic, social, and economic situations of each city. Similar to this study, assessing the characteristics of air pollution around megacities may contribute significantly to the development of city-based air quality policies to protect human health from PM and preserve the air environment. This study provides scientific references and baseline approaches for assessing air pollution characteristics in megacities worldwide.

## Conclusions

This study aims to assess the characteristics of the Seoul metropolitan area and Chungcheongnam-do region, classified based on the PM_10_ concentration variability. Cluster analysis of the average PM_10_ concentrations in three winters during 2018/19–2020/21 showed a total of four regional patterns, which differ from the administrative division. The obtained results demonstrated the effects of the locations of PM_10_ emission sources, topography, atmospheric chemical reactions, and airflow in the Korean Peninsula on the clustering results. The implemented policies in the study area to reduce air pollutants mostly focus on the management of emission sources at the administrative district scale. The present study suggests that the implementation of region-customized air quality management policies by the defined clusters is more effective in improving air quality compared to those implemented based on administrative districts.

## Data Availability

The data that support the findings of this study are available from the corresponding author, upon reasonable request.
